# Surface EMG-Based Hand Gesture Recognition Using a Hybrid Multistream Deep Learning Architecture

**DOI:** 10.3390/s26072281

**Published:** 2026-04-07

**Authors:** Yusuf Çelik, Umit Can

**Affiliations:** Computer Engineering Department, Munzur University, 62000 Tunceli, Turkey; ucan@munzur.edu.tr

**Keywords:** surface electromyography, hand gesture recognition, deep learning, biometric signal processing

## Abstract

**Highlights:**

**What are the main findings?**

- The suggested multistream hybrid model attained 96.4% accuracy and a 96.4% macro F1-score through the random-split with augmentation methodology, thereby exceeding previously documented results on the FORS-EMG dataset.
- The results indicate that the subject-wise procedure produced inferior performance on the FORS-EMG dataset.

**What is the implication of the main findings?**

- Hybrid deep learning models markedly enhance classification performance.
- The disparity in performance between the subject-wise and random-split procedures signifies that cross-user generalization continues to pose a challenge.

**Abstract:**

Surface electromyography (sEMG) enables non-invasive measurement of muscle activity for applications such as human–machine interaction, rehabilitation, and prosthesis control. However, high noise levels, inter-subject variability, and the complex nature of muscle activation hinder robust gesture classification. This study proposes a multistream hybrid deep-learning architecture for the FORS-EMG dataset to address these challenges. The model integrates Temporal Convolutional Networks (TCN), depthwise separable convolutions, bidirectional Long Short-Term Memory (LSTM)–Gated Recurrent Unit (GRU) layers, and a Transformer encoder to capture complementary temporal and spectral patterns, and an ArcFace-based classifier to enhance class separability. We evaluate the approach under three protocols: subject-wise, random split without augmentation, and random split with augmentation. In the augmented random-split setting, the model attains 96.4% accuracy, surpassing previously reported values. In the subject-wise setting, accuracy is 74%, revealing limited cross-user generalization. The results demonstrate the method’s high performance and highlight the impact of data-partition strategies for real-world sEMG-based gesture recognition.

## 1. Introduction

sEMG is a non-invasive biosignal acquisition technique that measures the electrical potentials generated during muscle contractions through electrodes placed on the skin surface. These signals represent motor commands transmitted by the nervous system to the muscles and directly reflect the musculoskeletal system’s functional state. sEMG data contain rich information about muscle force, activation duration, and motor unit characteristics [1,2]. Therefore, they are widely applied in domains such as human–machine interaction, neurological rehabilitation, prosthesis control, ergonomics, sports sciences, and biometric identification [2,3,4].

Recent advances in portable, wireless, and multi-channel recording systems have enhanced the applicability of sEMG not only in clinical settings but also in real-world conditions [1,5]. While temporal aspects of muscle activation, such as onset, duration, and intensity, can be directly measured, frequency-domain analyses enable more complex inferences, including muscle fatigue, neural control strategies, and functional asymmetries [2,6,7]. Owing to these properties, sEMG enables more in-depth analyses of neuromotor system functioning, thereby establishing itself as a valuable tool in both clinical and applied research contexts [1,7,8,9].

Recent studies have demonstrated that the high classification accuracies reported in controlled experimental settings often fail to generalize to real-world applications. This discrepancy is largely attributed to the inherent variability of sEMG signals across subjects, recording sessions, and data acquisition conditions [2,10]. For many years, meaningful information extraction from sEMG has primarily relied on feature engineering followed by classification. However, manual feature design has limited representational power, and inter-subject physiological variability often degrades performance in multi-user or cross-session scenarios [3,10]. In contrast, deep learning models and hybrid learning-based approaches that leverage time-domain representations or time–frequency transformations to learn richer temporal and spectral features from multi-channel signals have demonstrated strong performance in sEMG analysis [2,10,11].

On the other hand, a careful examination of the sEMG application domain and the deep learning literature reveals that developing models with higher classification accuracy alone is not sufficient. A major limitation is the scarcity of studies that systematically evaluate how robust and generalizable these models remain across variations in subjects, recording sessions, and data acquisition conditions, while explicitly accounting for protocol sensitivity. Achieving consistent predictions across users remains particularly challenging due to variability induced by factors such as anatomical differences, electrode placement, muscle activation strategies, fatigue, and acquisition settings [2,10]. In particular, generalizing to previously unseen users remains a significant bottleneck. Although approaches based on prototype learning, domain adaptation, and unsupervised transfer learning have shown promise in improving cross-subject performance, the reliability of subject-independent predictions remains limited [12,13,14]. Therefore, the key research gap in the literature is the lack of comprehensive studies that jointly and systematically investigate deep learning-based sEMG gesture recognition models not only in terms of within-dataset accuracy, but also with respect to cross-subject generalizability and sensitivity to evaluation protocols. This limitation highlights the critical need to learn effective gesture representations that remain robust to user and context variability and to validate them through protocol-aware, practically meaningful evaluation strategies [13,14].

In this study, a hybrid and multi-stream deep learning architecture is proposed to jointly model local, temporal, and global dependencies in sEMG-based gesture recognition tasks. The proposed framework integrates TCN, CNN, LSTM/GRU, and Transformer components to capture complementary feature representations at different levels. The effectiveness of the proposed model is extensively evaluated on the FORS-EMG dataset, where its impact on hand gesture recognition performance is systematically analyzed. Moreover, as a key contribution of this work, model performance is rigorously compared across different data partitioning strategies, thereby explicitly revealing the sensitivity of the results to the evaluation protocol. The findings demonstrate that integrating local feature extraction, temporal dependency modeling, and global contextual representation learning from multi-channel sEMG signals yields more consistent and generalizable performance, particularly in cross-subject scenarios.

This study focuses on sEMG-based gesture classification and positions its experimental framework as follows:

➢ We propose a gesture classification model that achieves high accuracy on the FORS-EMG dataset.
➢ The model is evaluated under both subject-wise and random-split protocols, allowing comparison between these two commonly used approaches.
➢ The experiments reveal that while random-split evaluation yields higher accuracy, the subject-wise protocol is critical for assessing cross-user generalization.

## 2. Related Work

A substantial body of research has been conducted on sEMG-based gesture recognition. These studies can generally be categorized according to the feature extraction techniques and classification models employed. The earliest line of work is characterized by handcrafted feature-extraction methods combined with classical machine-learning algorithms [15]. Within this group, gesture discrimination has been achieved using time-domain, spatio-temporal, wavelet-based, or hybrid descriptors. For instance, studies employing handcrafted features such as RMS, WL, and entropy-based measures combined with dimensionality reduction techniques and classical classifiers have demonstrated effective gesture recognition performance [16]. Such approaches offer advantages in terms of relatively low computational cost and interpretability of the extracted features. However, their strong dependence on the specific dataset has been shown to limit generalization performance across different users and varying data acquisition conditions [17,18,19,20].

In contrast, another line of research has focused on deep and hybrid architectures that learn feature representations directly from raw data. Within this paradigm, CNN-based models, time–frequency representation-driven approaches, multi-stream architectures, and attention-enhanced hybrid models have gained prominence. These models aim to more effectively capture both local patterns and long-range dependencies inherent in sEMG signals [21,22,23,24,25,26,27,28,29]. Furthermore, recent advances have explored neuromorphic computing paradigms, in which spiking neural networks (SNNs) are employed for EMG-based gesture recognition, enabling energy-efficient, low-latency processing suitable for real-time wearable and edge-computing applications [30]. Empirical findings indicate that learned feature representations often outperform traditional handcrafted feature-based approaches in many scenarios [31]. However, a considerable portion of the reported high performance in the literature is obtained under within-dataset or random-split evaluation settings, leaving unclear the extent to which these models generalize to previously unseen users. Recently, not only in sEMG-specific studies but also across the broader biosignal classification literature, transformer-based and hybrid architectures that integrate local and global modeling capabilities have become increasingly prominent. For instance, a recent survey by Anwar et al. [32] highlights the growing adoption of transformer-based models across various biosignal modalities for tasks such as classification, analysis, and physiological event detection. These models have been shown to effectively capture long-range dependencies through attention mechanisms and contextual learning. Similarly, Wang et al. [33] note that while most deep learning approaches in this domain rely on CNNs, RNNs, or their hybrids—where CNNs are effective at capturing local features and RNNs are often constrained by gradient-related limitations—temporal depthwise convolutional transformer architectures can model long-range dependencies with lower computational cost. In another study, Karnam et al. [28] demonstrate that a hybrid architecture combining CNN and BiLSTM components, capable of jointly learning inter-channel relationships and bidirectional temporal dependencies, yields strong performance in sEMG-based hand activity classification. Likewise, Yuan et al. [29] integrate time–frequency representations obtained via the Short-Time Fourier Transform (STFT) with CNN–BiGRU branches, followed by a transformer-based classifier, demonstrating that fusing spatial and temporal features can yield notable performance improvements. Overall, these developments indicate a clear trend toward hybrid transformer-based architectures that unify local pattern extraction, sequential dependency modeling, and global contextual representation within a single framework.

Studies based on approaches such as unsupervised transfer learning, prototype learning, and subject-independent classification have demonstrated that cross-subject performance can be improved through appropriate representation learning and adaptation strategies. Nevertheless, these works consistently emphasize that inter-subject variability remains a critical challenge for achieving reliable performance in real-world applications [12,13,14]. Moreover, other studies have shown that temporal variability across different recording days can further degrade model performance, highlighting the importance of cross-session and long-term evaluation in EMG-based systems [34]. In addition, recent studies have explored few-shot and class-incremental learning frameworks to address challenges related to limited data availability and evolving class distributions [35]. More importantly, recent benchmark studies such as EMGBench have shown that direct comparisons of results across different datasets and evaluation protocols can often be misleading, highlighting that out-of-distribution evaluation has become a central requirement for robust model assessment [4]. Furthermore, recent longitudinal datasets collected across multiple days have highlighted the importance of evaluating sEMG models under realistic conditions, demonstrating that both subject diversity and temporal variability are critical factors for robust and generalizable performance [36].

An examination of studies conducted on the FORS-EMG dataset reveals that both handcrafted feature extraction combined with classical machine learning methods and deep or hybrid architectures that learn representations directly from data have been widely employed. Early works primarily introduced the dataset and provided baseline comparisons, whereas subsequent studies have focused on improving performance through feature engineering and time–frequency-based deep representations [17,37,38]. However, studies that jointly address hybrid end-to-end representation learning together with protocol-aware evaluation on the FORS-EMG dataset remain limited. Overall, the literature indicates a clear evolution from handcrafted features to learned representations, and subsequently toward generalization-oriented approaches. Despite this progress, there remains a lack of studies that simultaneously consider hybrid end-to-end learning, cross-subject generalizability, and protocol-sensitive evaluation in the FORS-EMG context. Beyond achieving high accuracy, the present study aims to provide a deeper understanding of the conditions under which model performance remains meaningful and reliable.

## 3. Materials and Methods

This section describes the dataset used in this study, the preprocessing steps applied to the raw signals, and the experimental protocol adopted for training and evaluation.

### 3.1. FORS-EMG Dataset

In this study, we used the FORS-EMG dataset, which contains sEMG signals associated with hand and wrist movements. The dataset was collected at the Rajshahi University of Engineering & Technology, Bangladesh, from 19 healthy volunteers aged 25–40 years. Each participant performed twelve distinct movements under three forearm orientations (supination, neutral, and pronation). Every movement was repeated 5 times per orientation, yielding 180 recordings per subject and a total of 3420 sEMG signals.

The recordings were acquired using an eight-channel system at a sampling frequency of 985 Hz, with each trial lasting approximately 8 s. Surface electrodes were placed at two distinct locations on the forearm: four electrodes positioned circumferentially around the elbow and four around the mid-forearm, following the acquisition protocol described in [38]. This configuration enables the capture of muscle activity from both anterior (flexor) and posterior (extensor) muscle groups involved in hand and wrist movements. The signals were stored in MATLAB 2020a format as matrices of 8000 samples × 8 channels.

**Figure 1:**
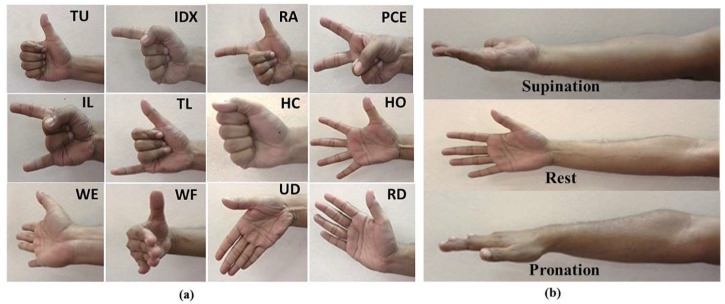
Twelve hand/wrist gestures and three forearm orientations are used in the FORS-EMG dataset. Panel (**a**) presents the visual representations of the gestures, while panel (**b**) shows the forearm positions used during data collection [38].

The dataset is organized into three forearm orientations. For each participant, recordings are separated into orientation-specific folders, and within each folder, repetitions of the corresponding movements are provided. According to the original experimental protocol [38], the dataset comprises recordings of 12 distinct gestures: thumb up, index extension, right angle, peace sign, index-little extension, thumb-little extension, hand close, hand open, wrist extension, wrist flexion, ulnar deviation, and radial deviation. To account for dynamic conditions, these gestures were executed across three forearm orientations: supination, neutral (rest), and pronation. These movements and orientations, as originally defined in [38], are illustrated in Figure 1.

The dataset, originally introduced in the study by Rumman et al. [38], has also been made publicly available on the Kaggle platform under the title “FORS-EMG: A Novel sEMG Dataset” [39]. In this study, the data were obtained from the Kaggle release.

### 3.2. Preprocessing

Before being used for model training, the raw sEMG signals were processed through a three-stage pipeline: filtering, segmentation, and normalization.

Filtering: To preserve the main frequency components of muscle activity while reducing noise, all channels were processed with a band-pass filter. Specifically, a 4th-order Butterworth band-pass filter in the 20–450 Hz range was applied. To avoid phase distortion, the forward–backward filtering method (filtfilt) was employed. The amplitude response of this filter can be expressed as Equation (1):

(1) $${\left|H\left(j\omega \right)\right|}^{2}=\frac{1}{\left(1+{\left(\frac{\omega }{\omega_{c}}\right)}^{\left(2n\right)}\right)},n=4$$

where $\omega_{c}$ denotes the cutoff frequency, and n is the filter order.

In practice, the filter was implemented as a digital 4th-order Butterworth band-pass filter, with normalized cutoff frequencies relative to the Nyquist frequency at a sampling rate of 985 Hz. Zero-phase filtering was performed using the filtfilt operation, which applies the filter in both the forward and backward directions, thereby eliminating phase distortion and preserving the signal’s temporal characteristics.

Segmentation: The filtered signals were divided into fixed-length windows. Each trial comprised approximately 7880 samples (≈8 s at 985 Hz), and the segment length was set to 492 samples (≈0.5 s). During segmentation, a 50% overlap (stride = 246 samples) was applied. With this method, 31 segments were generated per trial, resulting in approximately 106,020 segments for the entire dataset.

Normalization: To reduce amplitude variations across segments and to mitigate the impact of inter-subject variability, each segment was scaled using channel-wise z-normalization, as shown in Equation (2):

(2) $${Ẋ}_{\left(t,c\right)}=\frac{\left(X_{\left(t,c\right)}-\mu_{c}\right)}{\left(\sigma_{c}+\varepsilon \right)},X\in R^{\left(492\times 8\right)}$$

where $\mu_{c}$ and $\sigma_{c}$ denote the mean and standard deviation of channel c, respectively, and ε is a small constant added to ensure numerical stability. To prevent data leakage, normalization statistics (mean and standard deviation) were computed independently for each dataset split (training, validation, and test) and applied only within the corresponding split.

To enhance the model’s generalization capability and mitigate the risk of overfitting, a data augmentation pipeline was implemented across the defined experimental protocols. These augmentation techniques included time-shifting (within a range of ±8 samples), time-masking (randomly zeroing out segments of up to 28 samples with a probability of 0.25), and the addition of Gaussian noise (σ = 0.0025). These methods were applied to simulate temporal variations and sensor noise, ensuring the robustness of the hybrid architecture across different signal conditions. In all scenarios, data augmentation was applied exclusively to the training set, while the validation and test sets remained raw and unchanged. Furthermore, a baseline evaluation was conducted without any data augmentation to assess the intrinsic feature extraction capacity of the proposed architecture independently of data enrichment.

**Figure 2:**
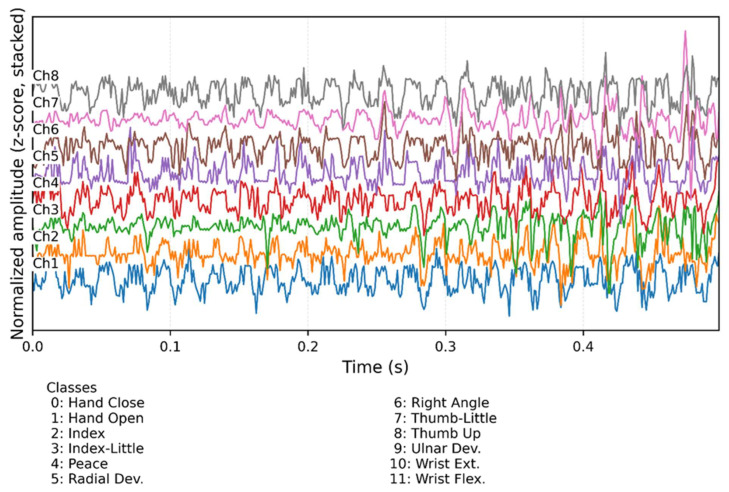
Example input segment from the FORS-EMG dataset. The upper panel shows eight normalized sEMG channels, while the lower panel illustrates the twelve hand and wrist gestures analyzed in this study.

To illustrate the structure of the input data used in this study, Figure 2 presents an example eight-channel segment from the FORS-EMG dataset along with the twelve hand and wrist gestures considered in this work.

### 3.3. Experimental Protocol

To prevent data leakage and to examine the effect of data augmentation techniques on model performance, three different dataset configurations were evaluated:

- Subject-wise split: To assess cross-participant generalization, the data from all 19 subjects were partitioned in a subject-wise manner. Seventeen subjects were used for training and validation, while the remaining two subjects were reserved for testing. Thus, the test set contained recordings unseen by the model during training. The train/validation split was stratified to preserve the class distribution.
- Random split with augmentation: Without separating participants, the data were randomly divided on a sample basis into 70% training, 15% validation, and 15% testing using stratified sampling. This scenario was designed to represent the upper-bound performance that can be achieved in practical applications. Both offline and online data augmentation methods were applied to the training set.
- Random split without augmentation: The same 70/15/15 stratified partitioning procedure was employed, but without applying any augmentation. This scenario was intended to isolate the effect of augmentation by providing a direct baseline.

To provide a more comprehensive evaluation, these configurations were designed to capture different aspects of model performance. The random-split settings offer a benchmark under less restrictive conditions and allow comparison with existing studies; however, they may introduce subject-dependent bias, as training and test samples are not fully independent at the subject level. In contrast, the subject-wise protocol provides a more realistic assessment of cross-user generalization. However, it should be noted that this evaluation is based on a limited number of unseen subjects, which may restrict the generalizability of the findings. This dual evaluation framework enables a clearer interpretation of the model’s behavior under both idealized and practical deployment scenarios.

### 3.4. Proposed Model

**Figure 3:**
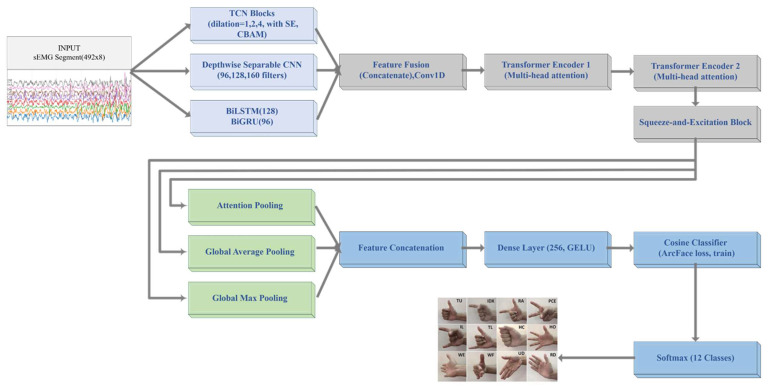
Overall architecture of the proposed multistream hybrid model for sEMG-based gesture classification. The model integrates three parallel streams—TCN blocks with SE and Convolutional Block Attention Module (CBAM) attention, depthwise separable CNN, and BiLSTM–BiGRU—followed by feature fusion, Transformer layers, pooling, and an ArcFace-based classification stage.

In this study, a hybrid deep learning architecture was developed to effectively capture the complex temporal and spectral characteristics of sEMG signals. The overall structure of the proposed model is presented in Figure 3.

In the TCN blocks, the input signals are first processed through three consecutive TCN blocks. These blocks employ increasing dilation rates (1, 2, 4) to capture long-term dependencies, while a squeeze-and-excitation (SE) mechanism rescales channel importance. In addition, a CBAM is integrated at the output of the TCN blocks, adding both channel and spatial attention and enabling the model to focus on the most relevant regions. The resulting representation is forwarded to the feature-fusion stage.

Depthwise separable convolution (DS-CNN), in a parallel stream, the input signals are processed by three consecutive depthwise separable convolutional blocks. Compared with standard convolutions, this design reduces the parameter count and computational cost while effectively capturing local time–frequency features.

In the third stream, the input signals are passed sequentially through bidirectional LSTM (BiLSTM) and bidirectional GRU (BiGRU) layers. This structure simultaneously learns forward and backward dependencies, thereby providing a richer representation of the signal’s dynamic properties.

The feature maps from the three parallel streams are concatenated, then passed through a 1D convolution and layer normalization. The fused representation is then processed by two successive Transformer encoder blocks, which leverage multi-head attention and feed-forward layers to capture global contextual information. CBAM is also integrated at this stage to further enhance channel- and spatial-wise attention.

At the final stage, the features are summarized using three complementary pooling strategies: attention pooling, which highlights information-dense regions via learnable attention weights; global average pooling; and global max pooling, which captures overall feature distributions. The three feature vectors are concatenated and projected into a 256-dimensional embedding layer, which is then passed to a CosineClassifier layer. Training is performed with the ArcFace loss function, which enhances inter-class separability while enforcing intra-class compactness.

For optimization, the AdamW algorithm is employed, with a learning rate dynamically adjusted using a warmup cosine-annealing schedule. To mitigate overfitting, dropout (0.3), L2 weight regularization, and stochastic weight averaging (SWA) are applied. In addition, hybrid loss variants (e.g., ArcFace combined with focal loss) are explored to further stabilize the model. The detailed hyperparameter configurations and specific settings used for the ArcFace-based optimization and SWA procedure are summarized in Table 1.

For optimization, the AdamW optimizer was employed with a weight decay of 3 × 10^−4^. The learning rate was scheduled using a warmup cosine-annealing strategy, with 5 warmup epochs. The final model was trained using the ArcFace loss function with scale parameter s = 30 and margin m = 0.20, combined with focal loss to improve class separability and stability. Stochastic Weight Averaging (SWA) was applied during the final stage of training by averaging the weights of the last 5 checkpoints, resulting in improved generalization and a more stable solution.

The proposed architecture was implemented using the TensorFlow v2.13 framework with the Keras API, and the training process was conducted on an NVIDIA Tesla A100 (Nvidia, Santa Clara, CA, USA) to ensure efficient convergence across all gesture categories.

In summary, the proposed model constitutes a multistream hybrid architecture that jointly captures both short-term local patterns and long-term global dependencies, thereby providing a robust framework for sEMG-based gesture classification.

## 4. Results

### 4.1. Evaluation Metrics

To evaluate the performance of the proposed model, standard metrics commonly used in multi-class classification problems were employed.

Precision: The proportion of correctly predicted positive instances among all positive predictions. As shown in Equation (3):

(3) $$Precision\;=\frac{\;TP\;}{\left(TP\;+\;FP\right)}$$

where *TP* denotes the number of true positives, and *FP* denotes the number of false positives.

Recall: Recall measures the proportion of actual positive instances that are correctly predicted. As defined in Equation (4):

(4) $$Recall\;=\frac{\;TP\;}{\;(TP\;+\;FN)}$$

where FN denotes the number of false negatives.

F1-score: The F1-score represents the harmonic mean of Precision and Recall, reflecting the balance between these two metrics. It is calculated as shown in Equation (5):

(5) $$F1\;=\;2\;\times \;\frac{(Precision\;\times \;Recall)\;}{\left(Precision\;+\;Recall\right)}$$

Accuracy: Accuracy represents the proportion of correctly predicted instances across all classes. It is defined in Equation (6):

(6) $$Accuracy\;=\;\frac{(TP\;+\;TN)\;}{\;(TP\;+\;TN\;+\;FP\;+\;FN)}$$

In addition, a confusion matrix was computed to analyze misclassifications in detail. The rows of the matrix represent the true classes, while the columns correspond to the predicted classes. The diagonal values indicate correct classifications, whereas the off-diagonal values represent misclassifications. This visualization highlights which class pairs the model most frequently confuses.

### 4.2. Experimental Findings

This section presents the performance results of the proposed model under three different experimental scenarios: (i) subject-wise split, (ii) random split without augmentation, and (iii) random split with augmentation. Model performance was evaluated using the metrics of accuracy, precision, recall, and F1-score. In addition, macro-average and weighted-average scores were reported to observe the effects of class imbalance. Table 2 presents comparative results for the proposed hybrid model across different data-splitting strategies.

Findings

- In the subject-wise scenario, the model achieved 74.0% accuracy and a macro F1-score of 73.7%, highlighting the challenge of generalization due to inter-subject variability.
- In the random split (no augmentation) scenario, accuracy increased to 92.9%, with F1-scores above 0.90 across all classes. This demonstrates the model’s strong discriminative capability when training and testing data originate from the same subjects.
- In the random split (with augmentation) scenario, accuracy reached 96.4% and the macro F1-score also reached 96.4%, indicating that augmentation techniques further improved the model’s generalization capacity.

The class-wise F1-scores of the proposed model under different data-splitting strategies are presented in Table 3.

Findings

- In the subject-wise split, Class 2 and Class 6 exhibited notably low performance (F1 < 0.55).
- In the random-split scenarios, F1-scores exceeded 0.90 across all classes.
- When augmentation was applied, F1-scores increased to approximately 0.95 for all classes, and variance across classes was minimized.

These results indicate that the subject-wise evaluation protocol, while realistic, remains highly challenging because the model struggles to generalize across individuals. By contrast, the random-split scenarios—especially with augmentation—yielded very high accuracy and stable performance. Therefore, in real-world applications, enhancing cross-user generalization will require testing on larger and more diverse datasets.

**Figure 4:**
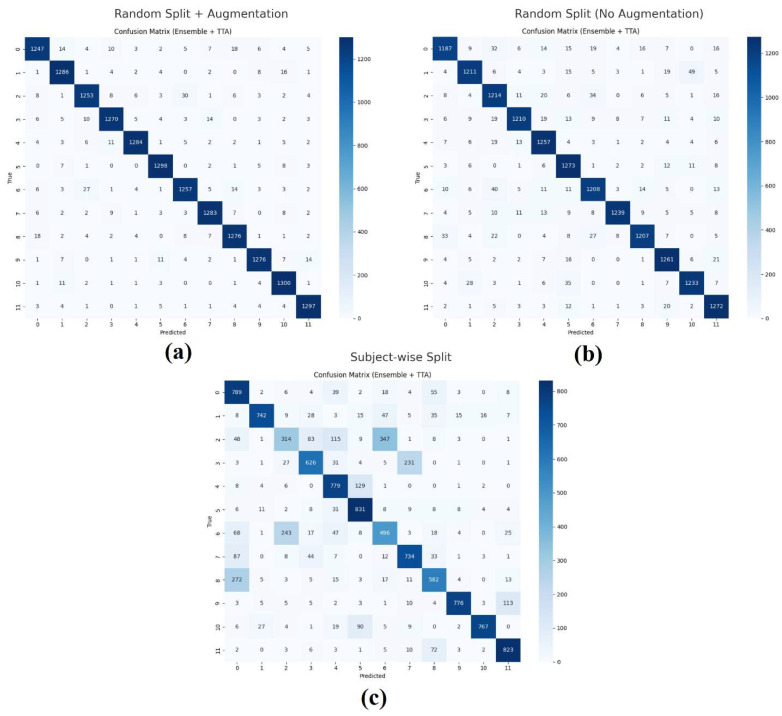
Confusion matrices of the proposed model under three experimental scenarios: (**a**) random split with augmentation, (**b**) random split without augmentation, and (**c**) subject-wise split.

Furthermore, the confusion matrices for the three experimental scenarios are presented in Figure 4. Figure 4a,b illustrate the high consistency of predictions in the random-split experiments, whereas Figure 4c highlights error clusters in the subject-wise case, particularly around Class 2 and Class 6.

A closer inspection of Figure 4c shows that the errors are concentrated in a few classes rather than being uniformly distributed. In particular, Class 2 (Index) and Class 6 (Right Angle) exhibit noticeably lower F1-scores and higher confusion with other classes.

These gestures are less distinctive and resemble several other hand movements, making them more difficult for the model to distinguish, especially under subject-wise conditions.

## 5. Discussion

The findings of this study reveal notable differences compared with previous work on the same dataset. In the original study by Rumman et al. [38], various classical methods were evaluated, with the best performance reported as an F1-score of 88.58% using LDA combined with SNTDF features. This result demonstrates that the FORS-EMG dataset constitutes a challenging benchmark due to inter-subject variability and the inclusion of three forearm orientations.

In their 2024 study, Aarotale and Rattani [17] evaluated three feature families (fused time-domain descriptors (fTDD), temporal-spatial descriptors (TSD), and discrete wavelet transform (DWT)) using both classical and deep learning methods. The highest accuracy, 94.95%, was achieved using TSD features combined with Random Forest, indicating that carefully engineered features can still provide strong performance, particularly in datasets with substantial orientation variability.

More recently, Aarotale and Rattani [37] transformed the signals into time–frequency images for fine-grained feature extraction. On the FORS-EMG dataset, this approach yielded accuracies of 93–94%. However, in certain configurations—especially when relying solely on time–frequency representations—the performance dropped to 56–60%. This finding suggests that orientation and user diversity in FORS-EMG may limit the effectiveness of purely time–frequency-based methods.

In contrast, the proposed hybrid model achieved an accuracy of 96.4% under the random-split-with-augmentation setting, outperforming the best previously reported results (94.95% for RF + TSD and 93–94% for XMANet). This result demonstrates that the proposed architecture is highly effective in capturing discriminative patterns in sEMG signals under conventional evaluation settings.

However, it is important to note that the performance obtained under the random-split-with-augmentation setting represents an upper bound on prediction performance and may be influenced by subject-dependent patterns. When evaluated under the more realistic subject-wise protocol, the accuracy dropped to 74%, indicating that the challenge of user-independent generalization remains unresolved. This discrepancy suggests that models trained using random splits may partially rely on user-specific signal characteristics rather than learning fully generalizable representations. Therefore, the findings highlight the importance of evaluation protocols and data partitioning strategies when assessing the practical applicability of sEMG-based gesture recognition systems. In addition, the subject-wise evaluation is limited to two unseen subjects, which may limit the strength of generalization claims. This limitation will be addressed in future work through more extensive cross-validation across larger subject groups.

It is important to note that most existing studies on the FORS-EMG dataset report results using random-split settings, which limit direct comparisons regarding real-world generalization. In this study, the inclusion of a subject-wise evaluation protocol yields a more comprehensive and realistic assessment than prior work. As shown in Table 4, the proposed model demonstrates competitive performance under the random-split setting, while clearly revealing the limitations in cross-user generalization under the subject-wise protocol. This observation highlights the critical role of data partitioning and evaluation strategies in interpreting model performance.

In addition, class-level analysis reveals that the model does not perform uniformly across all gestures. In particular, Class 2 (Index) and Class 6 (Right Angle) show lower performance compared to other classes, especially in the subject-wise setting. These gestures are less distinctive and resemble other movements, making them harder to distinguish and leading to greater confusion.

Most studies on sEMG-based gesture recognition have considered the human body under non-fatigued conditions. However, muscle fatigue is a critical physiological factor that directly affects sEMG signal characteristics. Kim et al. [40] demonstrated that muscle fatigue can be estimated from EMG signals using two frequency- and amplitude-based measures: zero-crossing rate (ZCR) and amplitude of muscle tension (AMT). Their findings experimentally confirmed that ZCR decreases while AMT increases under fatigue conditions. Based on these results, it has been suggested that muscle fatigue can influence classification performance. In another study, Ao et al. [41] compared the performance of traditional machine learning and deep learning models under fatigued and non-fatigued scenarios. Their experimental results showed that sEMG signal features such as root mean square (RMS) vary with fatigue, and these variations directly affect classification performance. These findings indicate that muscle fatigue is not only a physiological condition that can be monitored via EMG signals but also a factor that affects the consistency and generalizability of model performance. Although muscle fatigue was not explicitly considered as an independent variable in the FORS-EMG dataset used in this study, future work may incorporate this factor into evaluation protocols to achieve more realistic and robust assessments.

Although the proposed architecture is relatively complex, each component is designed to capture complementary aspects of sEMG signals, including local patterns, temporal dependencies, and global contextual relationships. However, a detailed ablation study was not conducted in this work, limiting the ability to quantify the individual contributions of each module. This is considered a limitation of the study and will be addressed in future work through systematic ablation analysis and model simplification.

## 6. Conclusions

This study proposed a multistream, hybrid deep-learning architecture for sEMG-based gesture classification on the FORS-EMG dataset. The proposed model, combining TCN blocks, depthwise separable CNN, BiLSTM–BiGRU, and Transformer layers, effectively captured both local and global dependencies.

Experimental results demonstrated that the model achieved 96.4% accuracy and 96.4% F1-score in the random-split-with-augmentation scenario, outperforming previously reported results under similar evaluation settings in the literature (e.g., 94.95% with RF + TSD, 93–94% with XMANet). However, in the subject-wise protocol, accuracy remained at 74%, highlighting that cross-user generalization remains a major challenge.

These findings underscore two main contributions of this work: (i) the development of a hybrid model that outperforms existing methods in terms of accuracy, and (ii) the explicit demonstration of the critical impact of data-splitting strategies on model performance. Future research should focus on improving user-independent generalization with larger and more diverse datasets, exploring domain adaptation and transfer learning approaches, and developing lightweight architectures suitable for deployment on embedded systems.

## Figures and Tables

**Table 1:** Summary of training hyperparameters and optimization settings for the proposed hybrid architecture.

Parameter	Value
Optimizer	AdamW (Weight Decay: 0.0003)
Learning Rate Schedule	Warmup-Cosine (5 warmup epochs)
Initial Learning Rate	0.0003
Batch Size	64
Total Epochs	90
Loss Function	ArcFace (with Hybrid Focal Loss)
Regularization	Dropout (0.3), L2, SWA

**Table 2:** Performance comparison of the proposed model under different data-splitting strategies. The results are reported as accuracy, macro-precision, macro-recall, and macro-F1 score.

Splitting Strategy	Accuracy	Macro Precision	Macro Recall	Macro F1-Score
**Subject-wise**	0.7401	0.7452	0.7401	0.7370
**Random Split (No Aug)**	0.9289	0.9296	0.9289	0.9289
**Random Split (With Aug)**	0.9638	0.9638	0.9638	0.9638

**Table 3:** Class-wise F1-scores of the proposed model under different data-splitting strategies. Results are reported for subject-wise, random split without augmentation, and random split with augmentation scenarios across all 12 gesture classes.

Class	Subject-Wise	Random Split (No Aug)	Random Split (with Aug)
**0**	0.7076	0.9141	0.9497
**1**	0.8583	0.9248	0.9633
**2**	0.4026	0.9003	0.9507
**3**	0.7126	0.9336	0.9614
**4**	0.7709	0.9349	0.9735
**5**	0.8207	0.9285	0.9759
**6**	0.5243	0.9148	0.9516
**7**	0.7501	0.9549	0.9676
**8**	0.6670	0.9306	0.9612
**9**	0.8864	0.9382	0.9670
**10**	0.8882	0.9341	0.9683
**11**	0.8546	0.9381	0.9748

**Table 4:** Comparison of the proposed model with previous studies conducted on the FORS-EMG dataset. The table summarizes methods, evaluation protocols, and reported performance values.

Study	Method	Evaluation Protocol	Performance
**Rumman et al. [38]**	LDA + SNTDF features	Random split	88.58% F1-score
**Aarotale & Rattani [17]**	TSD features + Random Forest	Random split (with augmentation)	94.95% Accuracy
**Aarotale & Rattani [37]**	Time–frequency images + XMANet	Random split (with augmentation)	93–94% Accuracy (best), 56–60% in some settings
**Our study**	Hybrid model (TCN+SE+ CBAM+Transformer, ArcFace)	Random split (with augmentation)	96.4% Accuracy, 96.4% F1-score
**Our study**	Same model	Subject-wise	74.0% Accuracy, 73.7% F1-score

## Data Availability

The dataset used in this study was obtained from the Kaggle platform. The dataset is a public dataset https://www.kaggle.com/datasets/ummerummanchaity/fors-emg-a-novel-semg-dataset (accessed on 12 January 2026).

## Abbreviations

The following abbreviations are used in this manuscript:

sEMG	Surface Electromyography
CNN	Convolutional Neural Network
GRNN	General Regression Neural Network
GRU	Gated Recurrent Unit
ViT	Vision Transformer
LSTM	Long Short-Term Memory
CWT	Continuous Wavelet Transform
LDA	Linear Discriminant Analysis
VMD	Variational Mode Decomposition
SVM	Support Vector Machine
CBAM	Convolutional Block Attention Module
TCN	Temporal Convolutional Networks
PCA	Principal Component Analysis
fTDD	fused time-domain descriptors
TSD	temporal-spatial descriptors
DWT	discrete wavelet transform
